# Improvise, Adapt, Overcome: How COVID-19 Transformed Inpatient Pediatric Gastroenterology

**DOI:** 10.1177/00099228211044854

**Published:** 2021-12

**Authors:** Daphne S. Say, Sabina Ali, Arvind Srinath, B. U. K. Li, Rajitha D. Venkatesh

**Affiliations:** 1 University of California, Davis, Sacramento, CA, USA; 2 University of California, Davis, Children’s Hospital, Sacramento, CA, USA; 3 University of California, San Francisco, San Francisco, CA, USA; 4 University of California, San Francisco, Benioff Children’s Hospital Oakland, CA, USA; 5 University of Pittsburgh, Pittsburgh, PA, USA; 6 UPMC Children’s Hospital of Pittsburgh, Pittsburgh, PA, USA; 7 Medical College of Wisconsin, Milwaukee, WI, USA; 8 Children’s Hospital of Wisconsin, Milwaukee, WI, USA; 9 The Ohio State University, Columbus, OH, USA; 10 Nationwide Children’s Hospital, Columbus, OH, USA

**Keywords:** telehealth, COVID-19, rounding, e-consults, inpatient, gastroenterology

## Abstract

The coronavirus disease-2019 (COVID-19) pandemic has disrupted inpatient pediatric services across the United States, creating opportunities for innovation. A recent Webinar organized by the Telehealth for Pediatric GI Care Now working group and sponsored by the North American Society of Pediatric Gastroenterology, Hepatology, and Nutrition provided insights into how inpatient pediatric gastroenterology services were affected and how physicians adapted during the crisis. These findings suggest the use of telehealth technologies may augment family communication and facilitate multidisciplinary care in the future. We anticipate that these innovative applications of telehealth will comprise a part of a toolkit for gastroenterologists to be used during this public health emergency and beyond.

The coronavirus disease-2019 (COVID-19) pandemic has greatly altered the daily lives of individuals and communities around the world. In the United States, many health care organizations and practices have responded to public health mandates by increasing hospital capacity to care for patients afflicted with COVID-19 while changing modalities of care delivery to reduce in-person contact.^1,2^ Inpatient censuses, particularly for pediatric units, decreased as “lockdown” orders were enacted in March to April 2020 across much of the United States.^3,4^ Pediatric gastroenterology (GI) inpatient services were not immune to these changes, with providers encountering new and unexpected challenges. Provider workforce composition changed considerably, with restrictions on trainee participation and reduced availability of attending personnel. Availability of personal protective equipment (PPE) and individual hospital COVID-19 screening policies also fundamentally limited the manner in which pediatric gastroenterologists care for patients admitted to the hospital.

We share findings gleaned from conference participants in our recent Telehealth for Pediatric Gastroenterology Webinar organized by the Telehealth for Pediatric Gastroenterology Care Now working group and sponsored by the North American Society for Pediatric Gastroenterology, Hepatology, and Nutrition on June 17, 2020. Attendees to a Breakout Session on Inpatient Management (35 participants) included gastroenterologists in academic and nonacademic practices, both with and without fellows. This diverse cohort of practitioners represented all regions of the United States, with the majority (22 participants, or approximately 63%) hailing from coastal, urban centers. Over half of attendees (20 participants, or approximately 57%) practiced at an academic medical center. Most of these academic practitioners (15 participants, or approximately 43%) represented institutions that sponsored GI fellowship training programs. The uneven initial impact of COVID-19 throughout the United States led to divergent iterations of inpatient pediatric GI services. Many physicians in hard-hit regions, like the New York metropolitan area, completely suspended all in-person patient services and provided all care through electronic modalities. Others chose to incorporate telehealth technology into their current inpatient practice. Use of telehealth applications allowed communication with caregivers who were unable to be at the bedside due to complying with social distancing rules while reducing strain on PPE resources. Additionally, many groups transitioned to “consult only” services, with pediatric hospitalists serving as the primary attendings of record during this period.

During our Breakout Session on Inpatient Telehealth, we conducted live polling of attendees on their personal experiences during the COVID-19 pandemic. We approached our webinar as a focus group interview, which provided in-depth exploration of the relatively unexplored topic of telehealth application to inpatient pediatric GI care. We then performed a simple designation analysis of our attendees’ responses.^5^ We viewed the increased frequency with which certain changes or ideas were noted (“mentions”) as indicative of importance and emphasis, allowing us to infer key trends from these data.

## Impact of Telehealth on Patient Care

**Figure 1.**
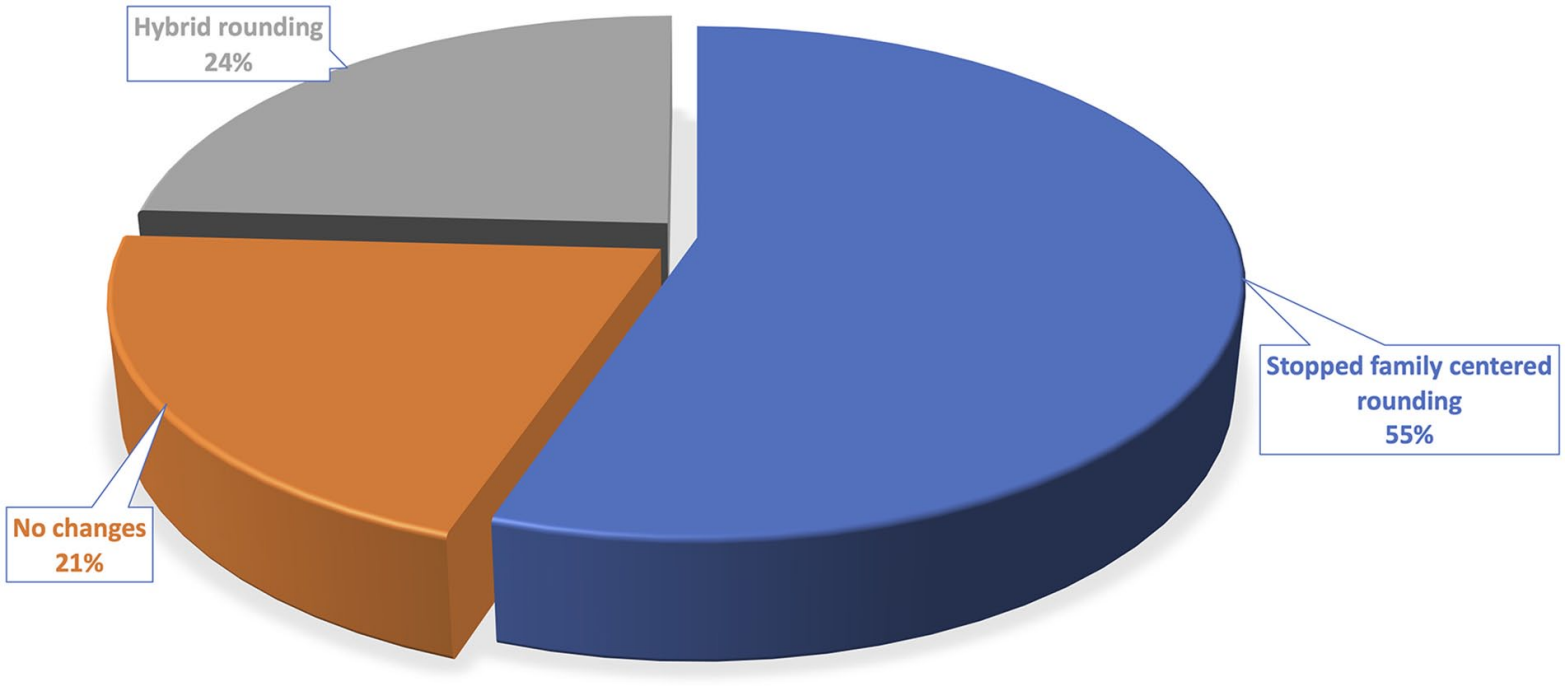
Respondent-reported modifications to inpatient rounding (total respondents, n = 35).

**Figure 2.**
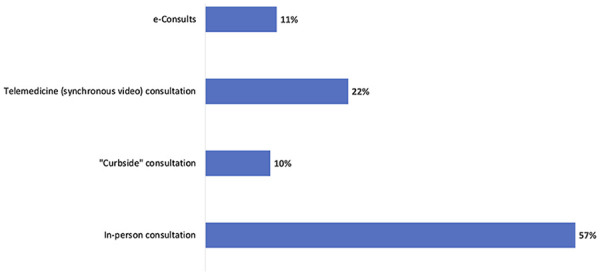
Respondent-reported modifications to inpatient and emergency department consultations (total mentions, n = 111).

**Figure 3.**
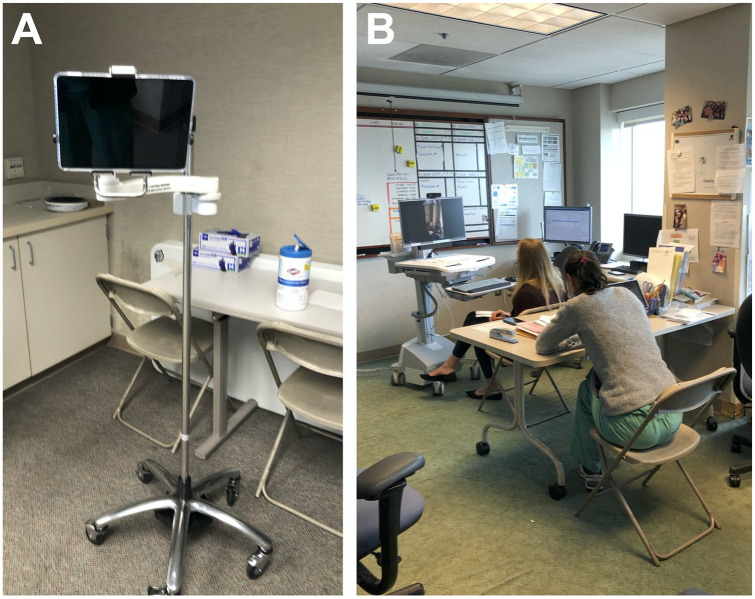
Adaptations to inpatient rounding at the University of California, Davis Children’s Hospital. (A) Apple iPad mounted on IV pole, utilized to enable rounding; (B) “Social distancing” rounds, with members of the inpatient team remotely connecting to the bedside.

Our attendees observed that the inpatient census for pediatric GI patients decreased considerably when compared with the pre-COVID-19 census, mirroring the decrease throughout the United States of total pediatric inpatient volume.^4,6^ For inpatient rounding, over 50% of attendees reported that they had stopped family-centered rounding (Figure 1). Those affected described the shift to either hybrid rounding (both in-person and tele-rounds) or tele-rounds only with the inpatient team. In addition, several attendees describe that, in an effort to concomitantly limit exposure to COVID-19 and preserve PPE supplies, they implemented creative and novel approaches to inpatient care delivery. Electronic consultations (e-Consults), consultative communications between providers occurring within a shared electronic health care record (EHR), have already been shown to be promising mechanisms to close care gaps in GI.^7^ The asynchronous nature of such programs allows referring providers to gather pertinent medical history, images, and pathology reports that are then sent to a specialist physician for diagnostic and treatment expertise. A notable proportion of attendees describe the use of these e-Consults, as well as synchronous telemedicine video encounters with the primary care provider present, as important pathways to provide inpatient care, including to patients in the emergency department (Figure 2). Physical examination of inpatients was typically limited to one provider (often the attending physician) to help reduce exposure to COVID-19. Many attendees developed unique methods to facilitate these in-person encounters, with use of tablet computers and other mobile devices to remotely connect other nonpresent members of the inpatient care team (Figure 3).^8^ For intensive care unit consults, the bulk of attendees continued to provide traditional in-person evaluations, but also reported use of telemedicine as well as “curbside” consultations. Several cited billing concerns as a barrier, as their institutions either did not have e-Consults available or were unsure on how to properly bill for them.

## Impact of Telehealth on Trainee Education

**Figure 4.**
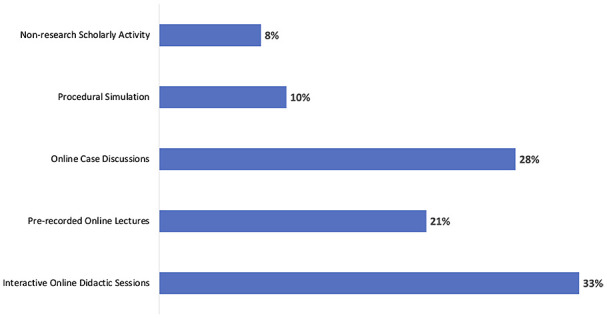
Respondent-reported modifications to educational approaches (total mentions, n = 75).

For those attendees working in institutions with trainees (namely, fellows and residents), precepting during the pandemic resulted in several changes. Most attendees reported precepting outpatient telemedicine visits either for the duration of the entire visit or, more often, only after the trainee had first evaluated the patient. For procedures, trainee participation was restricted due to concerns for exposure and limited PPE supply. Furthermore, fellows noted a significant decrease in procedure volume, attributed to decrease in overall patient volume and cancellation of elective procedures. This raised faculty attendees’ concerns regarding potential long-term effects on fellows’ procedural competency. Several attendees discussed the use of virtual lectures and case discussions to augment fellow learning, as well as incorporation of procedural simulation and nonresearch scholarly activities (Figure 4).

## Challenges Encountered and Future Directions

While there are several limitations to polling a limited number of pediatric gastroenterologists, the experiences of those in this Telehealth Webinar Breakout Session provided a window into the manner in which inpatient care was conducted during the COVID-19 pandemic. The multiple alterations to inpatient care during this crisis have reshaped the way in which patients and their families interact with their pediatric GI team. On the positive side, telehealth technology has enabled nonpresent family members, allied health providers, and other consultants to more easily participate in multidisciplinary care discussions, regardless of physical location. Webinar attendees noted new obstacles presented with the inclusion of telemedicine into daily inpatient practice. Coordination of telehealth meetings was time-consuming and required careful choreography to ensure patient privacy and seamless video connection. The lack of a traditional physical examination was disconcerting for many, with physicians citing decreased quality of patient interaction and increased anxiety regarding the potential for missed findings.^9^ Many attendees commented on the challenges of patient evaluation in the absence of key components of the gastroenterologist’s typical physical examination, like a digital rectal examination or abdominal palpation. Conversely, some shared that telehealth provided insight into the patient’s home environment, helping them understand how health concerns fit into the family’s overall priorities.

There remains much to be learned about the implications of this rapid and unprecedented shift to telehealth on the practice of inpatient pediatric GI. Though many webinar participants described feelings of apprehension regarding patient perception of remotely delivered care, the literature demonstrates that patients and families are generally quite satisfied with the use of telehealth, with convenience and ease of access to care cited as primary drivers.^10^ We, the authors, assert that the limitations on the traditional physical examination posed by the pandemic serve to emphasize the clinician’s critical need for an accurate, thorough history. We surmise that increased use of telehealth may compel providers to practice more defensively, by ordering more laboratory or imaging studies than they typically would as part of their evaluation. We also note that billing and reimbursement are likely to influence the use of telehealth both during the pandemic and after “shelter-in-place” restrictions have been lifted. We anticipate that future work on the impact of telehealth on physician behavior, financial concerns, and clinical outcomes will be needed to further evaluate these effects.

Our telehealth webinar highlighted several mechanisms by which technology can be used to augment inpatient services during the COVID-19 pandemic. The innovations developed to address the urgent need for physical separation comprise a series of tools that will remain part of the pediatric gastroenterologist’s repertoire, even after social distancing restrictions have been relaxed. Many providers and families appreciate the ability to participate in patient care and decision making, even when physically separated. We anticipate that the role of telehealth in patient care will continue to evolve, creating new opportunities as well as challenges for pediatric gastroenterologists to improve access and quality for patients and their families.

## Tips, Tricks, and Lessons Learned: A Summary of Inpatient Rounding Tactics Used During the COVID-19 Pandemic

- *Initial Approaches Deployed during the Surge*
  - Halted all in-person family-centered rounding
  - Transitioned to “consult only” service, with pediatric hospitalists assuming primary responsibility for GI patients (including those in the intensive care unit and emergency department)
  - Suspended all in-person GI services, with care provided only via electronic health modalities
- *Modifications to Inpatient Rounding*
  - Primary tele-rounds: GI team evaluated patient via telehealth only, with physical examination performed by an ancillary provider (eg, bedside nurse or mid-level provider)
  - “Hybrid” (in-person + telehealth component) rounds: attending gastroenterologist evaluated patient in-person and performed physical examination, while remainder of GI team participated via telehealth from outside the patient’s room
  - Consultation tele-rounds: pediatric hospitalist team evaluated patient in-person and performed physical examination, while GI team participated via telehealth in purely consultative capacity
- *Alternative Strategies for Provision of Consultative Care*
  - Synchronous video telehealth encounters with patient ± primary care provider, typically with documentation in EHR, usually billed
  - Asynchronous electronic communication with primary providers (e-Consults), typically with documentation in EHR, both billed and unbilled, depending on institution
  - “Curbside” consultation, typically unbilled

## Author Contributions

Drs Say and Venkatesh drafted the initial manuscript, created original figures and charts, analyzed poll data, and reviewed and revised the final manuscript. Drs Ali, Srinath, and Li reviewed the initial and final manuscript. Dr Li provided editorial guidance. All authors reviewed approved the final manuscript as submitted and agree to be accountable for all aspects of the work.

## References

[1] Berg EA Picoraro JA Miller SD , et al. COVID-19—a guide to rapid implementation of telehealth services: a playbook for the pediatric gastroenterologist. J Pediatr Gastroenterol Nutr. 2020;70:734-740. 32443021 10.1097/MPG.0000000000002749 PMC7273955

[2] Srinivasan M Asch S Vilendrer S , et al. Qualitative assessment of rapid system transformation to primary care video visits at an academic medical center. Ann Intern Med. 2020;173:527-535. 32628536 10.7326/M20-1814 PMC7370832

[3] Bosworth T. COVID-19 pandemic driving huge declines in pediatric service revenue. The Hospitalist. Published August 7, 2020. Accessed December 12, 2020. https://www.the-hospitalist.org/hospitalist/article/226657/pediatrics/covid-19-pandemic-driving-huge-declines-pediatric-service

[4] Wolfson BJ. Coronavirus pandemic hurting pediatric hospitals, too. Kaiser Health News. Published May 19, 2020. Accessed December 12, 2020. https://khn.org/news/the-pandemic-is-hurting-pediatric-hospitals-too/

[5] Krippendorff K Bock MA. The Content Analysis Reader. Sage; 2009.

[6] Hillstrom Z. Hospitals often lose money treating COVID-19 patients. The Colorado Springs Business Journal. Published August 27, 2020. Accessed September 2, 2020. https://www.csbj.com/premier/businessnews/healthcare/hospitals-often-lose-money-treating-covid-19-patients/article_f39203a4-e8ac-11ea-9c81-db42f95c5d0f.html

[7] Venkatesh RD Campbell EJ Thiim M Rao SK. e-Consults in gastroenterology: an opportunity for innovative care. J Telemed Telecare. 2019;25:499-505. 29973131 10.1177/1357633X18781189

[8] Siwicki B. Health system pieces together “virtual rounding” to cope with pandemic. Healthcare IT News. Published April 23, 2020. Accessed September 2, 2020. https://www.healthcareitnews.com/news/health-system-pieces-together-virtual-rounding-cope-pandemic

[9] Hyman P. The disappearance of the primary care physical examination-losing touch. JAMA Intern Med. 2020;180:1417-1418. 32832987 10.1001/jamainternmed.2020.3546

[10] Ramaswamy A Yu M Drangsholt S , et al. Patient satisfaction with telemedicine during the COVID-19 pandemic: retrospective cohort study. J Med Internet Res. 2020;22:e20786. 32810841 10.2196/20786 PMC7511224

